# The interest of gait markers in the identification of subgroups among fibromyalgia patients

**DOI:** 10.1186/1471-2474-12-258

**Published:** 2011-11-11

**Authors:** Bernard Auvinet, Denis Chaleil, Jean Cabane, Anne Dumolard, Pierre Hatron, Robert Juvin, Michel Lanteri-Minet, Yves Mainguy, Laurence Negre-Pages, Fabien Pillard, Daniel Riviere, Yves-Michel Maugars

**Affiliations:** 1 Locomotion Research Unit, Laval Hospital, 53015 Laval, France; 2 Department of Pharmacy, University Hospital, 49045 Angers, France; 3 Department of Internal Medicine, St Antoine Hospital, 75012 Paris, France; 4 Department of Rheumatology, University Hospital, 38043 Grenoble, France; 5 Department of Internal Medicine, University Hospital, 59000 Lille, France; 6 Pain Department, University Hospital, 06002 Nice, France; 7 Pierre Fabre Laboratories, Department of Clinical Research, 31319 Labège Innopole, France; 8 LN Pharma, Epidemiology, 31000 Toulouse, France; 9 Respiratory Functional Explorations, Department of Sport Medicine, University Hospital, 31059 Toulouse, France; 10 Department of Rheumatology, University Hospital, 44093 Nantes, France

## Abstract

**Background:**

Fibromyalgia (FM) is a heterogeneous syndrome and its classification into subgroups calls for broad-based discussion. FM subgrouping, which aims to adapt treatment according to different subgroups, relies in part, on psychological and cognitive dysfunctions. Since motor control of gait is closely related to cognitive function, we hypothesized that gait markers could be of interest in the identification of FM patients' subgroups. This controlled study aimed at characterizing gait disorders in FM, and subgrouping FM patients according to gait markers such as stride frequency (SF), stride regularity (SR), and cranio-caudal power (CCP) which measures kinesia.

**Methods:**

A multicentre, observational open trial enrolled patients with primary FM (44.1 ± 8.1 y), and matched controls (44.1 ± 7.3 y). Outcome measurements and gait analyses were available for 52 pairs. A 3-step statistical analysis was carried out. A preliminary single blind analysis using k-means cluster was performed as an initial validation of gait markers. Then in order to quantify FM patients according to psychometric and gait variables an open descriptive analysis comparing patients and controls were made, and correlations between gait variables and main outcomes were calculated. Finally using cluster analysis, we described subgroups for each gait variable and looked for significant differences in self-reported assessments.

**Results:**

SF was the most discriminating gait variable (73% of patients and controls). SF, SR, and CCP were different between patients and controls. There was a non-significant association between SF, FIQ and physical components from Short-Form 36 (p = 0.06). SR was correlated to FIQ (p = 0.01) and catastrophizing (p = 0.05) while CCP was correlated to pain (p = 0.01). The SF cluster identified 3 subgroups with a particular one characterized by normal SF, low pain, high activity and hyperkinesia. The SR cluster identified 2 distinct subgroups: the one with a reduced SR was distinguished by high FIQ, poor coping and altered affective status.

**Conclusion:**

Gait analysis may provide additional information in the identification of subgroups among fibromyalgia patients. Gait analysis provided relevant information about physical and cognitive status, and pain behavior. Further studies are needed to better understand gait analysis implications in FM.

## Background

In clinical trials and observational research studies, fibromyalgia (FM) is usually diagnosed according to American College of Rheumatology (ACR) 1990 criteria [1]. However, patients fulfilling the ACR classification criteria for FM do not constitute a homogeneous group and the classification of FM into different subgroups calls for broad-based discussion. Strazt et al [2] sought to identify different FM subgroups by distinguishing between FM with and without depression. Turk et al [3] showed that subgroups identified by cluster analysis, based on the Multidimensional Pain Inventory (MPI), could be applicable to FM patients; this subgrouping was based on psychosocial and cognitive characteristics. Giesecke et al [4] showed that the combination of these two features and pain sensitivity indices best distinguished subgroups of FM patients. Using the MPI, Thieme et al [5] identified three subgroups of FM patients on the basis of anxiety and psychiatric comorbidities. On the basis of the associated clinical signs and symptoms, Müller [6] found that primary FM can be divided into four subtypes: sensitivity to pain, comorbid pain-related depression, concomitant depression, and FM due to somatization. Recently, De Souza et al [7] featured two distinct subgroups using the Fibromyalgia Impact Questionnaire (FIQ): one was characterized by low anxiety levels, depression and morning tiredness while the other was characterized by elevated pain levels, fatigue, morning tiredness, stiffness, and depressive symptoms. Using the Medical Outcomes study 36-item Short Form Health Survey (SF-36), Oswald et al [8] detected two subgroups: the first one demonstrated psychological dysfunction, whilst the second achieved normal psychological scores. In a large survey, Wilson et al. [9] identified 4 subgroups of FMS patients by means of a cluster analysis based on 3 symptom factor scores: musculoskeletal symptoms, other physical symptoms, and cognitive/psychological symptoms. All mentioned studies illustrate the great interest of clinicians, in daily practice, in recognizing homogenous subgroups in FM to provide guidance for treatment decisions. Furthermore, research is ongoing to assess FM severity and subgrouping FM patients by means of quantitative measurements. More recently, Aparicio [10] found that handgrip was reduced in women with FM (p < .001), and inversely related to FM severity and symtomatology.

Gait analysis shows a growing interest in the neuropsychological domain, in particular with regard to cognition and dementia [11], as well as anxiety and depression [12]. The importance of cognition for gait became evident with the observation that frail or cognitively impaired elderly people could no longer walk while performing a secondary task such as talking [13].

Gait has to be considered as a cognitive function and not as an automated motor task [14]. Walking at a self-selected speed requires cognitive resources, particularly executive function and attention [15]. Given that FM patients have cognitive function impairment that could mimic about 20 years of ageing [16] we hypothesized that gait could be impaired in FM patients. Affect also has to be taken into account; indeed, depression and anxiety have a negative influence on gait, possibly by reducing attention to gait control [17].

In a preliminary study, we showed that gait impairment in FM patients affects walking speed and walking-derived markers such as stride frequency (SF), stride regularity (SR), and cranio-caudal power (CCP) which measures the kinesia of the movement according to the cranial-caudal axis [18]. Furthermore, Heredia Jiménez [19] found a significant difference between FM women and control groups as regard spatial-temporal parameters of gait. In addition, correlations were found between FIQ and spatial-temporal parameters of gait in FM [19]. Therefore, we decided to undergo a case-control observational study on the interaction between FM and gait in primary non-depressed FM patients without any neurological treatment.

Our study aimed at characterizing gait disorders in primary FM patients, looking for correlations between gait markers and main FM features, and subgrouping FM patients according to gait markers.

## Methods

### Study Design

This multicenter study was an exploratory, observational open trial enrolling outpatients with primary FM and healthy control subjects matched for age, height, and weight. The one-to-one matching was realized by each participating center. Ethics approval for this study was obtained from the Human Ethics Committee of Nantes University. All participants provided informed written consent according to Helsinki declaration.

### Study selection criteria

Healthy control subjects and patients were women aged 20 - 55 years able to walk 40 meters without assistance. Healthy control subjects, if possible without any concomitant medications, underwent a complete physical examination. Patients with primary FM were recruited according to ACR criteria. All of them had achieved an average pain visual analogue score (VAS) of at least 40 on a scale of 0-100 mm, over the past week. They presented non-major depression according to the Mini-International Neuropsychiatric Interview (MINI) [20].

Patients and controls with any of the following conditions were excluded from the study: current or past cardiovascular, pulmonary, neurological, rheumatologic, endocrine, digestive or renal diseases; current systemic infection; active cancer; and any history of/or existing musculoskeletal disorders that could induce walking disability such as obesity (BMI > 30 kg/m^2^), pregnant or nursing women, or current psychiatric illnesses.

A routine work-up was performed at a central laboratory for all FM patients. Normal results constituted one of the study inclusion criteria.

### Study procedure

Data from a previous study [18] were used to calculate the study power and the sample size. Gait markers differed considerably and were of clinical value in 14 FM patients and 14 matched controls. Thus a sample size of 22 subjects in each group would provide statistical power of > 0.95 with an alpha level of 0.05 to assess physical functions by gait analysis in FM patients and matched control subjects. No information was available for the subgrouping of patients by gait analysis. Based on this data, we planned to include 60 patients and 60 controls. Study participants were screened/selected at the time of a first visit (V1). Baseline assessments and gait tests were realized at the second visit (V2), which took place between one and four weeks later, depending on the wash-out period needed for discontinuing central nervous system (CNS) active drugs such as antidepressants, antiepileptics, mood stabilizers, centrally acting muscle relaxants, hypnotics, and analgesics type II or III. Patients' consent for drugs withdrawal was included in their informed written consent. When patients experienced an exacerbation of their FM pain severe enough to require additional analgesia, paracetamol was allowed as a rescue analgesic. Likewise, low doses of benzodiazepines were allowed in case of anxiety.

### Assessment tools

At V1, demographic data, exercise status, lifestyle, physical examination, vital signs and patients' biological tests were obtained. In addition, three psychological assessments were carried out: the MINI, the State-Trait Anxiety Inventory (STAI) and the Beck Depression Inventory (BDI). Only certain parts of the MINI questionnaire were used, particularly those exploring major depression, generalized anxiety, panic disorder, agoraphobia, social phobia, obsessive compulsive disorder, post-traumatic syndrome, alcohol and substances abuse and psychotic disorders [20]. The STAI was used to assess state and traits of anxiety [21]. The BDI was used to quantitatively assess depression symptoms of patients [22]. Pain during the previous week was evaluated at V1 and V2 by means of the 100-mm VAS pain score. VAS at V1 and V2 were similar so we referred to VAS at V1. Baseline assessments included the Short Form McGill Pain Questionnaire (SF-MPQ) [23]. The Coping Strategies Questionnaire (CSQ) was used to assess patient's strategies to cope with chronic pain, and the efficacy of these strategies in controlling pain [24]. The CSQ assessed the use of 6 cognitive coping strategies (diverting attention, reinterpreting pain sensations, coping self-statements, ignoring pain sensations, praying or hoping, catastrophizing) and 1 behavioral coping strategy (increasing activity level). Fatigue intensity was measured by the Chalder Fatigue Scale (CFS) [25]. Sleep quality and disturbances were assessed by the Pittsburg Sleep Quality Index (PSQI) [26]. The SF-36, including both physical component summary (PCS) and mental component summary (MCS), was utilized for the assessment of health status, functional status and quality of life [27]. The FIQ was used to assess the overall symptomatology of FM patients [28].

### Gait analysis

The gait analysis system (Locometrix™) includes an accelerometric sensor, a recording device and a computer program for processing the acceleration signals. The sensor, composed of three accelerometers, is incorporated into a semi-elastic belt, which is fastened around the subject's waist. One accelerometer is aligned with the cranio-caudal axis of the body. A gait test was performed during a stabilized walk at a self-selected speed, which allowed obtaining stabilized gait measurements. Patients and controls wore their usual walking shoes. The duration of gait analysis was long enough to include 19-21 gait cycles.

### Gait markers

The analysis software allowed the characterization of gait by measuring the following markers: walking speed, SF, stride length, right and left step symmetry, SR, and CCP. Walking speed is measured in meter per second (m/s), and SF is the number of gait cycles per second (Hertz (Hz)). Stride length is calculated from the average speed divided by the SF (meter (m)). Right and left step symmetry on vertical accelerations is an index of overall symmetry (dimensionless). SR quantifies the spatial-temporal similarity between successive gait cycles, which is a measure of stride-to-stride variability (dimensionless). CCP (W/kg) measures the amount of movement (magnitude and frequency) in the cranio-caudal axis and can be considered as a measurement of the kinesia [29].

### Statistical analysis

The primary assessment was the quantitative evaluation of gait markers. A preliminary single blind analysis was performed as an initial validation of gait markers. The statistician had to cluster all participants (patients and controls, identities were coded) into FM patients and control subjects using k-means cluster analysis limited to two clusters. Then the descriptive analysis was performed on each gait marker alone and then by combining different markers in order to improve the sensitivity or specificity i.e. the ability to detect FM or controls. Comparisons between FM patients and control subjects were made in an open statistical analysis. The purpose was to quantify FM patients according to psychometric, self-questionnaire assessment and gait markers. Main outcomes were obtained using all items, which differed significantly between patients and controls without any center effect. In addition, ROC curve analysis of gait markers was performed. Finally, correlations between gait markers and main outcomes were calculated.

Thirdly, using the hierarchical cluster analysis and calculating the Euclidian distances between groups according to Ward, we described subgroups for each gait marker and looked for significant differences in self reported assessments.

## Results

### Population characteristics

Out of 132 subjects (73 FM patients and 59 controls) eligible to be enrolled at V2, 104 (52 matched pairs) completed the study according to the protocol. Table 1 shows that patients and controls characteristics at V1 were not statistically different except for professional status, concomitant treatment, STAI-trait, and BDI. The STAI-state was not different between patients and controls. No pain was recorded in the control group. Self-report measures at V2 are shown in table 2. The variance analysis showed a center effect for physical functions in FIQ (*P *< 0.05), and reinterpreting pain sensations in CSQ (*P *< 0.05). These 2 items were therefore excluded from the statistical analysis. Gait markers in patients and controls, and variance analysis of the center effect are shown in table 3. Consequently, 3 gait markers were selected for statistical analysis: SF, SR and CCP. ROC curves confirmed the utility of gait markers in the identification of patients (area under the curve for SF, SR and CCP were 0.740 (0.044), 0.678 (0.052) and 0.690 (0.053), respectively). The blind cluster analysis showed that SF was the most discriminating marker among patients and controls (38/52 (73%)).

**Table 1:** Characteristics of the study population at the time of the screening visit

Measurements	FM patients n = 52	Controls n = 52	*P*
Age, mean (SD) years	44.1 (8.1)	44.5 (7.3)	0.59
Height, mean (SD) cm	165 (5.8)	164 (6.4)	0.35
BMI, mean (SD) k/m^2^	24.2 (4.1)	23.8 (4.4)	0.95
Marital Status			0.57
Married, n	33	38	NS
Other situations, n	19	14	NS
Education level			0.97
Primary education, n	2	2	NA
Secondary education, n	27	26	0.98
Higher education, n	23	24	0.98
Professional Activity, n	50	32	< 0.0001
Physical exercise, n	32	32	NS
Concomitant treatment, n	37	17	< 0.0001
BDI, mean score (SD)	15.8 (8.2)	3.8 (3.7)	< 0.0001
STAI-State, mean score (SD)	37.3 (13.6)	29.6 (8.4)	0.16
STAI-Trait, mean score (SD)	44.1 (12.6)	35.6 (9.0)	0.02
VAS weekly, mean score (SD)	63.4 (21.5)	2.8 (7.8)	0.0001
Tender points, mean (SD)	16.2 (2.1)	0	NA
Pain duration, mean (SD) years	7.3 (6.6)	0	NA

NS = not stated; NA = not applicable; BDI = Beck Depression Inventory; STAI = State-Trait Anxiety Inventory; VAS = Visual Analog Scale.

**Table 2:** Self-report measurements at the time of baseline visit

Measurements	FM patients n = 52	Controls n = 52	*P*
VAS weekly, mean score (SD)	70 (18.1)	3.6 (9.7)	< 0.0001
SF-MPQ, mean score (SD)	24.5 (9.4)	1.3 (3.3)	< 0.0001
FIQ, mean score (SD)	56.6 (15.1)	4.5 (8.3)	< 0.0001
CSQ			
Diverting attention, mean score (SD)	16 (7.4)	10 (9.3)	0.008
Reinterpreting pain sensations, mean score (SD)	9.2 (7.7)	5.5 (6.4)	0.01
Coping self-statement, mean score (SD)	24.2 (7.1)	16.5 (10.6)	0.0009
Ignoring pain sensation, mean score (SD)	15.6 (8.1)	13.5 (9.3)	0.48
Praying and hoping, mean score (SD)	12.1 (7.2)	6.8 (7.5)	0.0002
Catastrophizing, mean score (SD)	14.5 (9.2)	4.6 (5.7)	< 0.0001
Increasing activity level, mean score (SD)	18.8 (6)	14.3 (9.6)	0.09
CFS, mean score (SD)	20.8 (5.6)	11.3 (2.2)	< 0.0001
PSQI, mean score (SD)	11.3 (4.3)	4.3 (2.7)	< 0.0001
SF-36			
Physical component summary, mean score (SD)	34 (7.5)	56 (4)	< 0.0001
Mental component summary, mean score (SD)	40.6 (10.5)	50.9 (6.7)	0.002

VAS = Visual Analog Scale; SF-MPQ = Mc Gill Pain Questionnaire; FIQ = Fibromyalgia Impact Questionnaire; CSQ = Coping Strategies Questionnaire; CFS = Chalder Fatigue Scale; PSQI = Pittsburg Sleep Quality Index; SF-36 = Short Form 36 items Medical Outcomes Study Questionnaire

**Table 3:** Gait markers, center effect, and ROC curve analysis in the study population

Gait markers	Patients n = 52	Controls n = 52	P	Center effect	Area under the ROC curve
Walking speed, mean (SD) (m/s)	1.18 (0.19)	1.32 (0.17)	0.00007	*P *< 0.03	ND
Stride Frequency, mean (SD) (Hz)	0.93 (0.07)	0.99 (0.07)	0.000008	*P *= 0.36	0.740 (0.044)
Symmetry, mean (SD) (WD)	213 (39)	227 (38)	0.067	*P *< 0.03	ND
Stride regularity, mean (SD) (WD)	267 (38)	293 (39)	0.0007	*P *= 0.10	0.678 (0.052)
Cranio-caudal Power, mean (SD) (W/Kg)	2.59 (1.45)	3.63 (1.51)	0.0005	*P *= 0.12	0.690 (0.053)

ND = not done; WD = without dimension

### Correlation analysis

No correlation was found between gait markers and SF-MPQ, CFS, PSQI, SF-36. A tendency to correlation was shown between SF-36 PCS and SF (*P *= 0.06). VAS was negatively correlated to CCP (r = -0.33, *P *= 0.01). The FIQ score was negatively correlated to SR (r = -0.34, *P *= 0.01), and had a tendency to correlate with SF (r = -0.26, *P *= 0.06). Among CSQ items, only diverting attention and coping statement were positively correlated to SF. Coping self statement and catastrophizing were positively and negatively correlated to SR, respectively (Table 4).

**Table 4:** Correlations between gait markers and main outcomes measurements

Outcomes measurements	Gait markers		
	
	Stride frequency	Regularity	CCP
VAS weekly pain			r = -0.33, *P *= 0.01
FIQ score	r = -0.26, *P *= 0.06	r = -0.34, *P *= 0.01	
CSQ score			
Diverting attention	r = 0.42, *p *= 0.002		
Coping self-statement	r = 0.28, *P *= 0.04	r = 0.31, *P *= 0.03	
Catastrophizing		r = -0.27, *P *= 0.05	
SF-36 (PCS)	r = 0.27, *P *= 0.06		

CCP = Cranio-caudal Power; VAS = Visual Analog Scale; FIQ = Fibromyalgia Impact Questionnaire; CSQ; Coping Strategies Questionnaire; SF-36 (PCS) = Short Form 36 items Medical Outcomes Study Questionnaire- physical component summary

### Cluster analyses

The cluster analysis of SF (Table 5) allowed the identification of 3 distinct subgroups with an Euclidian dissimilarity scale of 17 (F ratio = 76, *P *= 0). The SF mean standard error (SE) for subgroups I (n = 9), II (n = 24) and III (n = 19) was 1.04 (0.01), 0.94 (0.007), and 0.86 (0.008), respectively. No differences were observed for BDI, STAI-trait, SF-MPQ, CSQ, CFS, PSQI, SF-36 MCS subscore and SR. The following differences have been observed between these 3 subgroups: VAS score was significantly lower in subgroup I than in subgroup II (*P *= 0.01). FIQ score was significantly lower in subgroup I compared to subgroups II and III (*P *= 0.004). PCS subscore of SF-36 was significantly higher in subgroup I than group III (*P *= 0.04), and CCP score was significantly higher in subgroup I than the other two subgroups (*P *= 0.0002).

**Table 5:** Cluster analysis of stride frequency and variance analysis of main outcomes measurements in fibromyalgia patients

	Subgroup I n = 9	Subgroup II n = 24	Subgroup III n = 19	Comparison of subgroups	F ratio	P
SF, mean (SE) (Hz)	1.04 (0.03)	0.94 (0.02)	0.86 (0.02)	I, II ≠ III	76	0
VAS weekly, mean score (0 - 100)	56.9 (12.9)	76.9 (7.1)	67.5 (8.1)	I ≠ II	4.99	0.01
FIQ, mean score (0 - 100)	42.0 (10.6)	59.6 (5.8)	59.8 (6.6)	I ≠ II & III	6.20	0.004
SF-36 (PCS), mean score (0 - 100)	39.9 (5.8)	33.5 (3.0)	32.2 (3.4)	I ≠ III	3.35	0.04
CCP, mean (W/Kg)	4.2 (1.0)	2.6 (0.5)	1.9 (0.6)	I ≠ II & III	10.63	0.0002

SF = stride frequency; SE = standard error; VAS = Visual Analog Scale; FIQ = Fibromyalgia Impact Questionnaire; SF-36 (PCS) = Short Form 36 items Medical Outcomes Study Questionnaire - physical component summary; CCP = Cranio-caudal power

Cluster analysis of SR (Table 6) allowed the identification of 2 distinct subgroups with a dissimilarity scale of 17 (F ratio = 88, *P *= 0). The mean SR ± standard error for subgroup I (n = 32) was higher than for subgroup II (n = 20) 291 ± 4 versus 229 ± 5. No difference was observed for VAS, SF-MPQ, CFS PSQI, SF-36, SF and CCP. Patients with low SR were more depressed (*P *= 0.03) and showed more anxiety traits (*P *= 0.03) than patients with normal SR. Furthermore, patients with low SR had reduced coping strategies such as self-statement (*P *= 0.003) as well as greater tendency towards catatrophizing (*P *= 0.002). Finally, low SR was associated with higher FIQ score (*P *= 0.02).

**Table 6:** Cluster analysis of stride regularity and variance analysis of main outcomes measurements in fibromyalgia patients

	Subgroup I n = 32	Subgroup II n = 20	F ratio	P
SR, mean (SE) (WD)	291.3 (8.4)	229.3 (10.8)	88	0
BDI, mean score (0 - 63)	13.8 (2.8)	19.0 (3.7)	5.35	0.03
STAI-Trait, mean score (20 - 80)	41.2 (4.4)	48.9 (5.7)	4.97	0.03
CSQ self-statement, mean score (0 - 36)	26.5 (2.4)	20.7 (3.1)	9.49	0.003
CSQ catastrophizing, mean score (0 - 36)	11.6 (3.1)	19.3 (4.0)	10.18	0.002
FIQ, mean score (0 - 100)	52.8 (5.2)	62.7 (6.8)	5.74	0.02

SR = Stride regularity; SE = standard error; WD = without dimension; BDI = Beck Depression Inventory; STAI = State-Trait Anxiety Inventory; CSQ = Coping Strategies Questionnaire; FIQ = Fibromyalgia Impact Questionnaire

Cluster analysis of CCP values (results not shown) allowed the identification of 3 distinct subgroups with a dissimilarity scale of 17 (F ratio = 184, *P *= 0). The mean CCP (SE) was higher in subgroup II (n = 8) than subgroups I (n = 24) and III (n = 20): 5.31 (0.18) versus 1.41 (0.10) and 2.92 (0.1), respectively. On the other hand, the multivaried analysis of these subgroups, based on main outcome measurements, showed no differences between the 3 subgroups, except for VAS that was significantly low in the subgroup II compared to subgroups I and III: 51.9 versus 72.2 and 74.6, respectively, *P *= 0.006.

## Discussion

### Patients

Only patients with primary FM were enrolled in the study. Excluded patients were those with secondary fibromyalgia in relation to an underlying disease such as rheumatoid processes that could interfere with gait analysis. All study patients fulfilled the ACR criteria and presented primary FM without major depression based on the MINI questionnaire. However, 3 patients had BDI scores reaching the value of severe depression [30]. This could be due to the fact that total BDI score may give a misleading impression of the nature and degree of affective disturbances in chronic pain, in relation with BDI items assessing physical symptoms [31]. In our study, we found a strong positive correlation between BDI scores and somatic disturbances subscores. According to FIQ values few of our patients have severe FM with a FIQ score over 70 [32]. This finding could be related to the absence of patients with major depression (according to the MINI) as well as obese patients. Depression constitutes one of the items measured by the FIQ. Obesity may contribute to the severity of FM as it reduces physical functioning and increases fatigue [33], which are a part of some FIQ items. However, no significant association between FM symptoms and obesity was found in the same study. On the other hand, obesity was associated with significant gait abnormalities such as lower SF (p = 0.01), decreased SR (p < 0.001), and reduced CCP (p < 0.001) in comparison with controls (unpublished data). So FM obese patients were excluded in this study.

### Gait assessment

Ambulatory gait analysis has been demonstrated as a reliable method in clinical practice for outpatients, [34]. Such gait analysis systems has been designed for clinicians looking for quantifying gait abnormalities, to grade gait disorder-related disability, and to provide a better pathology's understanding for tailored treatment. This way of thinking about gait analysis was emerged from previous researches conducted in gait analysis laboratory, including FM patients [35]. Nevertheless, gait analysis has to be considered as a complementary exam. The gait analysis system, gait tests and derived markers have been previously validated [36,37]. Walking at self-selected overground speed is the gold standard of gait analysis [38]. SF expresses basic rhythmic stepping while SR expresses gait unsteadiness according to temporal and dynamic parameters, which is referred to as gait variability [39]. CCP, previously studied in Parkinson's disease (PD), is correlated to motor score and proposed as a measurement of kinesia [29].

### Gait markers in fibromyalgia

In our study, SF revealed to be the best gait marker differentiating FM patients from controls allowing the identification of 3 out of 4 subjects in each group. This finding raises the question of the importance of SF in FM, and its significance in terms of underlying mechanism, contrary to PD patients in whom SF remained unaltered [40]. In addition, SF in our study was correlated to diverting attention and coping self assessment, which are associated with high physical and low psychosocial disability levels [41]. Finally, we found a weak correlation between SF, FIQ and physical component of SF-36. These results suggest that SF may be of interest in assessing the physical component of FM.

In our study, SR was strongly correlated to FIQ and to catastrophizing that is a major CSQ item. In FM, catastrophizing is a main cognitive factor, and can prospectively predict high level of pain and depression, and low quality of life [41]. SR measures the unsteadiness of gait, and it has been linked to many neurological diseases such as PD [29], Alzheimer's disease [42], and preclinical stages of dementia [12]. Interestingly, a recent investigation disputed the concept of automatic regulation and suggested that stride time variability is related to specific cognitive processes, namely executive function and attention [39]. Therefore, SR could be suggested as a measurement of cognitive reserve in FM.

CCP, which measures kinesia, was found to be the only correlation in our study between pain and gait analysis. In fact, CCP measurement reflected the fear of pain (kinesiophobia), which is a classic behavior in FM patients leading to a reduction in their movements [43]. This result highlights the significance of gait analysis and pain behavior assessment in chronic painful conditions previously described in patients with lower back pain [44]. Kinesia evaluation by means of CCP measurement could be a promising area of research in the field of pain behavior management.

Gait markers were not correlated to fatigue, sleep disorders or mental component of SF-36. This result has to be discussed according to the gait test that was primarily designed to analyze gait in the basal condition at a self-selected speed. Additional components to the gait test could thus be suggested such as 6-minute walk test or dual task in order to consider fatigue and sleep disorders in FM patients.

### Cluster analysis

The cluster analysis strengthened the clinical significance of each gait marker. Cluster analysis of SF identified an interesting subgroup characterized by a normal SF associated with low pain level (VAS), reduced overall symptomatology (FIQ), high activity (PCS) and hyperkinesia. The two other subgroups with reduced SF were characterized by high pain level, low activity and hypokinesia.

Cluster analysis confirms the correlations observed in the subgroup with low SR, characterized by reduced self-statement, increasing catastrophizing, and high FIQ. This subgroup was also distinguished by more anxiety and depression. Therefore, one may raise the question of possible overlap between the subgroup with low SR, identified on the basis of gait analysis, and other subgroups distinguished by means of different cluster methods based on anxiety, depression and cognitive features. Consequently, considering the important role of affect and cognition in FM patients, one could suggest SR measurement before initiating cognitive-behavioral therapies in order to adapt treatment approaches to patients' characteristics [45]. Cluster analysis of CCP enabled the identification of a subgroup with low pain level and hyperkinesia. Our hypothesis is that each gait marker is correlated to one of the major traits of FM patients such as pain (CCP), physical activity (SF), and catastrophizing (SR).

### Clinical value

The three main gait markers (SF, SR, CCP) were correlated to some major clinical characteristics of patients (VAS, FIQ score, Coping self statement, Catastrophizing, PCF from SF-36). Such statistical results are needed, but are not sufficient for clinical applications. This point received a first answer through Cluster analysis. Each cluster analysis of gait markers provided distinct subgroups in term of mean value of the gait marker taken into account, but homogenous in term of clinical characteristics. Furthermore a preliminary study showed that the improvement of gait markers is of clinical significance in FM patients after a 12-week rehabilitation and exercise training program [46].

### Study limitations

Our study has some limitations. The presence of a center effect concerning some gait markers such as step length and gait speed, on the one hand, and physical activity subscore of FIQ, on the other hand, did not permit the determination of correlations between these items. The reduced speed in FM [18,19] could be more difficult to interpret because it is the product of SF by SL. The assumption that SL is supraspinally controlled by phasic output from basal ganglia to the supplementary motor area should be discussed, not only according to grading and subgrouping of FM but also according to the disease's underlying mechanisms. Another limitation is the absence of FM patients having major depression. Currently, it is demonstrated that depression is associated with gait deterioration and cognitive impairment [47]. Further studies are required in order to better understand gait disorders in FM patients with depression. As well, the exclusion of obese patients from the study population could be considered as a study limitation, however it is important to take into account that the prevalence of obesity varies greatly between the different studies according to the area of the study. For instance, in North America a prevalence of 47% was reported [33] while in North Europe the prevalence is only of 10%. [48]. Further limitations are related to sample characteristics. Our study involved only women, since the occurrence of FM in men is fairly limited.

## Conclusions

In conclusion, gait marker measurement, in particular SF, SR, and CCP, could be of interest in subgrouping FM patients, with no major depression. In our study, SF was the most sensitive and specific gait marker for distinguishing FM patients from controls. High SF values characterized a hyperkinetic subgroup of FM patients. So, subgrouping FM patients on the basis of SF measurement could facilitate the prescription of physical activity for patients with normal SF, and incite clinicians to prescribe a slowly progressive physical activity program for those with reduced SF. SR allows the identification of two subgroups, which were significantly different in terms of coping, affective status, and overall symptomatology (FIQ). Reduced SR values characterized a subgroup of FM patients with poor affect and cognitive abilities. SR measurement could therefore help in the orientation of the behavioral therapy in this subgroup of patients. Due to the strong correlation between CCP and pain level, we suggest that CCP measurement could be of interest in the assessment of pain behavior among FM patients. Further studies are needed to assess the interest of gait markers in the identification of subgroups of FM patients.

## Competing interests

This work was supported in part by a grant from Pierre Fabre Laboratories. B.A. received research support form Pierre Fabre Laboratories. Y.M. is an employee and shareholder of Pierre Fabre Laboratories. L.N.P. received honoraria from Pierre Fabre Laboratories. All other authors have declared no conflicts of interest.

## Authors' contributions

BA conceived the idea of the study. BA, YM, YMM conceived the project. YM and LNP wrote the protocol and conducted the coordination of the trial. DC designed and performed the statistical analysis. BA and DC wrote the manuscript. All authors contributed to the study design, provided input into the writing of the protocol, were involved in data collection, provided feedback on drafts of this paper, read, and approved the final manuscript.

## Pre-publication history

The pre-publication history for this paper can be accessed here:

http://www.biomedcentral.com/1471-2474/12/258/prepub
